# Advances in Percutaneous and Endovascular Locoregional Therapies for Primary and Metastatic Lung Cancer

**DOI:** 10.3390/cancers18081189

**Published:** 2026-04-08

**Authors:** Maria Mihailescu, Adam G. Fish, David C. Madoff

**Affiliations:** 1 Department of Radiology, University of Pittsburgh Medical Center, 200 Lothrop Street, Pittsburgh, PA 15213, USA; mihailescum3@upmc.edu (M.M.); fishag@upmc.edu (A.G.F.); 2 Section of Interventional Radiology, Department of Radiology and Biomedical Imaging, Yale School of Medicine, 330 Cedar Street, New Haven, CT 06510, USA; 3 Section of Medical Oncology, Department of Internal Medicine, Yale School of Medicine, New Haven, CT 06520, USA; 4 Section of Surgical Oncology, Department of Surgery, Yale School of Medicine, New Haven, CT 06520, USA

**Keywords:** lung cancer, locoregional therapy, thermal ablation, microwave ablation, cryoablation, image-guided ablation, ablative margins, bronchial artery chemoembolization, oligoprogression, salvage therapy, robotic-assisted ablation, electromagnetic navigation

## Abstract

Many patients with primary or metastatic lung cancer are not candidates for surgery, additional radiation, or further systemic therapy due to advanced age or comorbidities; this creates a need for minimally invasive locoregional options. Image-guided thermal ablation (IGTA) is being applied across a broader spectrum of lesions, while bronchial artery chemoembolization (BACE) is emerging as a therapy option for treatment-refractory advanced disease. Recent studies in thermal ablation have focused on optimizing energy delivery and protocols, as well as improving ablation zone predictability and analysis. Advances in lesion targeting, including cone beam CT fusion, electromagnetic guidance, and robotic-assisted ablation, allow for treatment of subcentimeter and ground-glass lesions in anatomically challenging locations. Growing clinical experience supports IGTA for intrathoracic oligoprogression and as salvage therapy after recurrence. In the endovascular space, improved imaging, microcatheters, and drug-eluting microspheres have expanded the use of BACE for disease and symptom control in advanced lung cancer. Multimodal strategies combining minimally invasive locoregional treatments with systemic therapies and radiation are being explored, with early data showing improvements in survival without increased toxicity. This narrative review synthesizes emerging techniques, clinical data, and indications for percutaneous and endovascular lung cancer treatments and underscores the need for prospective and randomized trials to refine patient selection, treatment sequencing, and long-term outcomes.

## Simple Summary

Many patients with lung cancer cannot undergo surgery, additional radiation, or further drug therapy, leaving them with limited treatment options. This review examines recent advances in image-guided thermal ablation and bronchial artery chemoembolization; two minimally invasive therapies used for lung cancer treatment. Image-guided thermal ablation uses extreme heat or cold delivered through percutaneously placed probes to destroy tumor tissue, while bronchial artery chemoembolization delivers chemotherapy directly to the tumor through its blood supply. Advances in energy delivery, navigation, and post-treatment imaging assessment have expanded the range of tumors that can be treated safely and effectively. This review also highlights emerging evidence for combining locoregional therapies with immunotherapy, radiation, and chemotherapy to improve outcomes beyond what can be achieved with a single treatment modality alone. The advances described in this review support a greater role for minimally invasive therapies in multidisciplinary lung cancer care.

## 1. Introduction

Lung cancer remains the leading cause of malignancy-related mortality worldwide with approximately 2.5 million new cases a year and 1.8 million deaths in 2022 [1]. As the prevalence of smoking declines, adenocarcinoma has surpassed squamous cell carcinoma as the dominant histological subtype. This shift is also attributed in part to non-tobacco risk factors such as air pollution, radon, and second-hand smoke [1,2,3]. These non-tobacco exposures are recognized as major risk factors among never-smokers, who represent 15–20% of lung cancer cases globally [1]. With an aging and growing population, lung cancer incidence is projected to increase to 4.6 million cases a year by 2050 [1]. At the same time, wider adoption of low-dose CT screening and advances in cross-sectional imaging have also increased detection of early non-small cell lung cancer (NSCLC) and limited metastatic disease [4].

Surgical resection remains the standard curative treatment for early-stage NSCLC, and stereotactic body radiotherapy (SBRT) is an effective non-surgical alternative. For locally advanced or metastatic disease, systemic chemoimmunotherapy regimens guided by molecular biomarkers, such as EGFR, ALK, and PD-L1, have significantly improved survival in locally advanced or metastatic disease [3,5]. However, approximately 70% of lung cancer patients are not surgical candidates due to age, comorbidities, or low pulmonary reserve, and many ultimately have local recurrence, oligoprogression, or treatment-limiting toxicity with SBRT or systemic therapy [4,6,7]. As the global burden of lung cancer grows, so will the population of patients requiring treatment alternatives beyond surgery, radiation, and systemic therapies. Image-guided thermal ablation (IGTA) is a lung-sparing local treatment option that can be repeated and is particularly valuable for medically inoperable patients, those with limited pulmonary reserve, or management of oligoprogressive disease [5]. Recent advancements in energy delivery platforms, intraprocedural imaging, navigation systems, and ablation zone assessment have expanded the spectrum of lesions that can be treated percutaneously with reliable tumor coverage. In the endovascular space, new catheter technology and drug-eluting microspheres have improved the safety and technical success of bronchial arterial chemoembolization (BACE), allowing for its use for patients with advanced lung cancer who cannot tolerate or have exhausted other therapy options. Emerging combination strategies pairing thermal ablation and BACE with chemotherapy or immune checkpoint inhibitors are also being explored to prolong disease control and improve symptom burden.

In this review, we provide an updated overview of IGTA and BACE in lung cancer, with a focus on technology-driven developments, outcomes, and expanding applications. We draw from peer-reviewed studies identified through targeted PubMed searches using a combination of terms related to lung cancer epidemiology, percutaneous thermal ablation, image guidance and navigation, ablative margins, bronchial artery chemoembolization, and multimodal treatment strategies including ablation combined with immunotherapy, radiation, and chemotherapy. An emphasis was placed on studies published between 2020 and 2026. Clinical practice guidelines and reference lists were also used to identify additional relevant sources. This is a narrative review, and a formal systematic search protocol was not used.

## 2. Energy Delivery and Ablation Zone Optimization

### 2.1. High-Power Microwave Ablation

Microwave ablation (MWA) offers several advantages as a locoregional therapy for lung tumors when compared with RFA, including shorter procedure times, larger and more predictable ablation zones, less sensitivity to tissue impedance, and relative resistance to heat sink from adjacent vessels [7,8,9,10]. Despite the central role of power selection in achieving adequate ablation margins, power settings remain largely operator-dependent, with current practice often relying on vendor-specific charts that do not reflect the aerated perfused lung environment [11,12,13]. Key clinical studies discussed in this review are summarized in Table 1.

The microwave ablation treatment of lung tumors (MALT study) was one of the first prospective multicenter efforts to standardize lung MWA power–time protocols based on tumor size (up to 4 cm) and origin (primary versus metastatic) [14]. The pre-defined wattage (W) and time settings were technically successful and showed reproducible ablation zones across centers. With power escalation for larger or primary tumors, local tumor progression (LTP) rate did not increase with greater tumor size, suggesting that appropriately scaled power can maintain disease control even in tumors up to 4 cm [14].

However, the relationship between power and local control remains unresolved, with subsequent studies reaching different conclusions. At power levels ranging from 20 to 65 W to achieve technical success (coverage of lesion with 5 mm margins), local recurrence (LR) was associated mainly with lesion size and location (central versus peripheral) rather than power or ablation time. This suggests that once adequate margins are obtained, additional energy does not further improve tumor control [15]. A separate multicenter analysis found that standard power (≥60 W) was associated with greater time to LR compared with low power (<60 W), with the strength of this association varying by tumor histology [7]. In contrast, a multicenter propensity-weighted comparison of 40 W and 50 W MWA, in which 40 W was the default and escalation was reserved for challenging tumors or incomplete margins, showed superior complete ablation rate at 12 months and fewer complications in the lower-power group [9].

The safety implications of higher power are also inconsistent between studies; while some studies show no clear association between power level and complication rates [15], others identify maximum power as an independent predictor of pleural effusion, pulmonary hemorrhage, hemothorax, and pneumothorax [26,27]. Overall, current evidence supports a default low-power strategy with selective escalation guided by tumor characteristics such as histology and intraprocedural margin assessment [9,14,15,26,27]. Prospective multicenter randomized trials incorporating standardized margin endpoints and power escalation criteria are needed to safely extend ablation to larger and more aggressive tumors.

### 2.2. Cryoablation Protocol Refinements

Cryoablation has unique advantages, including real-time visualization of the ice ball for margin assessment and preservation of adjacent tissue architecture compared with heat-based modalities [4]. However, similar to MWA, protocols vary with respect to number and duration of freeze–thaw cycles and use of active versus passive thawing [16].

**Figure 1:**
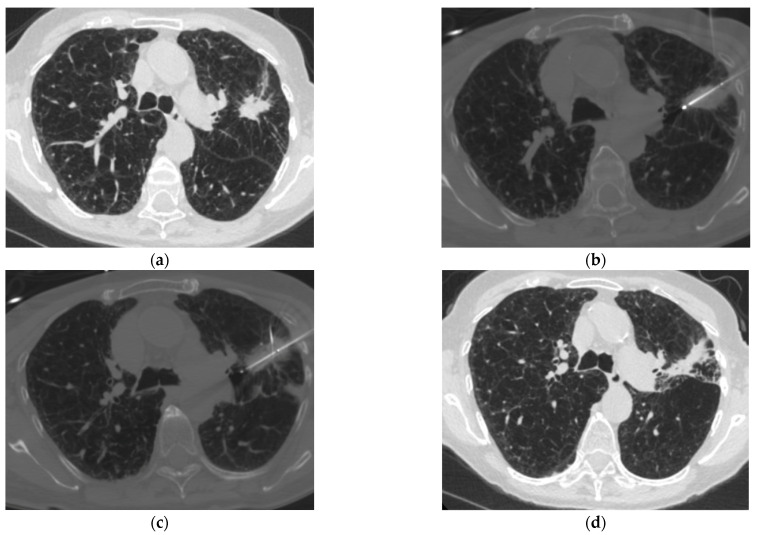
CT-guided percutaneous cryoablation of previously irradiated left upper lobe lung cancer. A 70-year-old female with history of left upper lobe (LUL) cancer status post-radiation presented for cryoablation of enlarging LUL nodule. (**a**) Pre-operative CT demonstrates moderate-to-severe upper lobe predominant emphysema with biapical pleuroparenchymal scarring and diffuse bronchial wall thickening. A 2.5 × 2.1 cm spiculated LUL lesion represents the recurrence. (**b**,**c**) Three cryoprobes were positioned in the lesion and a triple-freeze protocol was performed (3 min freeze, 3 min thaw, 8 min freeze, 5 min thaw, 8 min freeze). (**d**) A 3-month post-ablation CT shows increased consolidation and volume loss in the treated LUL, consistent with expected post-cryoablation changes.

Available evidence, synthesized in a systematic review and meta-analysis of 19 observational studies, supports a triple freeze–thaw cryoablation protocol, incorporating a short first freeze, longer subsequent freezes, and active final thaw to maximize tumor control and reduce risk of hemoptysis [16]. While protocol parameters of triple freeze–thaw cycles and active thaw were associated with superior LTC in univariate analysis, tumor size was the only factor independently associated with LTC in multivariate meta-regression [16]. This suggests that the benefit of protocol optimization is limited by tumor size; a separate cohort study found that ending triple freeze–thaw protocol with active thaw was associated with significantly lower hemoptysis rates without compromising LTC [28]. Figure 1 illustrates a CT-guided triple freeze–thaw protocol using three probes to treat a recurrent lesion in previously irradiated lung.

The available data are largely retrospective with heterogeneous lesions and follow-up. Standardized reporting of technical parameters is also needed to allow cross-study comparison [16]. Randomized controlled trials designed to assess freeze–thaw protocols are required to define the optimal cryoablation approach in lung cancer.

Transbronchial cryoablation is also being explored as an approach that may reduce risk of pneumothorax and pleural hemorrhage, particularly for central lesions. An exploratory study using a novel 1.9 mm flexible cryoprobe with navigational bronchoscopy and cone beam computed tomography (CBCT) guidance achieved 100% technical success in nine peripheral stage IA lung cancers and metastases, with complete ablation in seven of nine tumors at 6 months and no pneumothorax or hemorrhage [29]. At present, transbronchial cryoablation remains investigational pending larger studies with longer follow-up.

### 2.3. Ablation Zone Optimization

Achieving adequate ablative margins while minimizing collateral injury remains a central technical challenge in percutaneous lung ablation. Variability in ablation geometry and limitations of margin prediction contribute to inferior LTC compared with SBRT [30]. A fundamental limitation of ablation planning is its reliance on manufacturer geometric charts derived from homogeneous, unperfused animal tissue. These do not account for patient-specific factors such as tissue heterogeneity, location, or lung aeration, all of which influence ablation geometry [13,30].

In MWA-treated lung lesions, actual ablation zones exceeded vendor predictions along the long axis by 5 mm but were 6 mm narrower than predicted along the short axis, with the discrepancy being amplified at higher energies [13]. This asymmetric distortion means that reliance on manufacturer charts may simultaneously overtreat in one dimension while undertreating in another.

Computational models that integrate tumor dimensions, probe position, surrounding tissue composition, adjacent vessels, and background lung properties from pre-treatment CT offer a potential solution [30]. Compared with vendor charts, one such model substantially reduced ablation zone surface error (3.65 ± 1.12 mm) and volume overprediction (31.5% for the computational model versus 141% for the geometric model) [30]. A transition towards patient-specific computationally predicted ablation zones is a key technological advancement to improve margin predictability, but current models are largely tested on retrospective datasets. Prospective validation integrating computational prediction into pre-procedural planning is needed to determine if this approach translates to better LTC.

## 3. Advances in Image Guidance and Navigation

Needle precision and trajectory execution determine both technical success and complications in percutaneous IGTA. Conventional CT guidance is limited by in-plane needle advancement, lack of real-time three-dimensional feedback, and the need for repeated manual adjustments, particularly when targeting small or anatomically challenging lesions. Recent advances in intraprocedural imaging and navigation aim to address these limitations.

### 3.1. Cone Beam CT with Image Fusion

Although most percutaneous lung ablations are performed with standard CT guidance, access to dedicated interventional CT is limited at many centers. CBCT with image fusion provides intraprocedural three-dimensional guidance registered to fluoroscopy, allowing trajectory planning, real-time needle visualization, and confirmation of ablation coverage [6]. In a large retrospective cohort, CBCT-based augmented fluoroscopy was combined with intraparenchymal fine needle adjustment (IPFA), a technique that employs a mounted pneumatic arm to allow for submillimeter needle adjustments within the lung. This workflow substantially improved diagnostic yield of subcentimeter lesions (10.1% to 50.0%, *p* < 0.001) and was associated with a lower pneumothorax rate, likely reflecting fewer pleural passes [6].

### 3.2. Electromagnetic Navigation-Assisted Ablation

Electromagnetic navigation (EMN) uses an end-expiration pre-procedure CT to generate a three-dimensional virtual anatomic map registered to the patient via fiducial markers. An electromagnetic field generated around the patient enables continuous real-time tracking of the needle on this map, facilitating execution of challenging trajectories that would be difficult under conventional CT guidance. Respiratory phase tracking can be incorporated to synchronize needle advancement to end-expiration, reducing image misregistration from respiratory motion [31]. In a prospective study of 48 pulmonary nodules including 21 pure ground-glass nodules (GGNs) (mean lesion diameter of 15.4 ± 5.9 mm), EMN-assisted biopsy with MWA achieved 95.8% diagnostic yield with a mean targeting error of 1.84 ± 1.08 mm [31]. MWA was technically successful in all lesions with no local recurrence at one year and pneumothorax and hemoptysis rates of 14.6% [31]. This compares favorably with a meta-analysis of CT-guided biopsy of lung nodules ≤ 20 mm, which reported 90% diagnostic accuracy with higher complication rates [32]. These results are limited to a single-center single-arm experience; comparative studies with longer follow-up are required to establish the role of EMN-assisted percutaneous biopsy and ablation in clinical practice.

### 3.3. Robotic-Assisted Ablation

Needle manipulation within lung parenchyma is a major contributor to procedure-related complications, most commonly pneumothorax [6,33]. Robot-assisted ablation addresses this by enabling precise execution of planned trajectories, including out-of-plane approaches, while minimizing needle adjustments [33]. In a prospective single-center study comparing robotic-assisted RFA of pulmonary metastases against a matched retrospective CT-guided freehand cohort, the robotic approach achieved 100% technical success, with significantly fewer needle manipulations (median 0 versus 4.5, *p* < 0.001) and adverse events (AEs) (*p* = 0.01) [33]. Accurate execution of out-of-plane trajectories was highlighted as a key advantage. However, robotic platforms require general anesthesia and specialized equipment, with current lung data being limited to a single modality at one center. An ongoing clinical trial of robot-assisted percutaneous lung ablation of GGNs (NCT07105813) may clarify its role in early lung cancer management.

These three technologies expand the range of treatable lesions by allowing precise targeting of small and anatomically challenging tumors that would be difficult or unsafe to treat under conventional CT guidance. For all platforms, the primary mechanism of benefit appears to be reduction in needle manipulation and pleural passes, suggesting that improved first-pass accuracy regardless of specific navigation technology is the key driver of minimizing procedure-related complications. Initial data points towards clear improvements in intraprocedural visualization, real-time tracking in challenging anatomy, and minimizing parenchymal manipulation. Comparative studies across platforms with standardized reporting of accuracy, complication rates, and long-term outcomes are needed to define the optimal navigation approach for each clinical context.

## 4. Ablative Margins and Immediate Imaging Assessment

### 4.1. Ablative Margins and Local Control

Across all percutaneous lung ablation modalities, the ability to achieve adequate circumferential ablative margins is a primary modifiable determinant of LTC. For NSCLC, a margin of ≥10 mm is generally recommended, and the American College of Chest Physicians notes that margins > 8–10 mm are associated with lower risk of recurrence [34,35]. When larger margins are not feasible, margins ≥ 5 mm produce significantly better complete ablation rates than 1–4 mm [36]. Prospective clinical data reinforce the margin-outcome relationship: in 45 biopsy-proven IA NSCLC lesions (median size 2 cm) treated with RFA, each 1 cm increase in largest axial diameter of ablation zone at three-month follow-up reduced the odds of two-year recurrence by 52% (*p* = 0.02) [37].

This relationship is also the basis for current tumor size limitations in IGTA, with National Comprehensive Cancer Network (NCCN) guidelines supporting its use for NSCLC tumors < 3 cm, as smaller tumors are more likely to be completely encompassed by the ablation zone [3]. Given the central role of margins in oncologic outcomes, reliable intraprocedural and early post-ablation margin adequacy assessment is critical.

### 4.2. Immediate Imaging Markers and Recurrence Risk

Contrast-enhanced CT at approximately one-month post-ablation remains the standard for evaluating treatment response. However, this approach has an inherent limitation of precluding same-session retreatment. Intra-procedural or immediate post-ablation imaging addresses this gap by stratifying recurrence risk and identifying cases for additional same-session ablation.

For heat-based modalities, a circumferential ground-glass opacity band around the lesion is an early marker of effective treatment, whereas focal thinning or interruption of this halo has been linked to subsequent sites of local recurrence [38]. These observations are consistent with pathology showing microscopic tumor involvement extending 6–8 mm beyond the visible tumor edge [38]. Cryoablation has an inherent advantage in margin assessment as its thermal margins are directly visualized throughout the procedure, allowing for real-time shaping of ablation geometry [4].

CT densitometry offers complementary prognostic information beyond margins alone. Higher Hounsfield units (HUs) within the ablation zone and smaller pre- to post-procedure HU differences are associated with subsequent local progression [37,39,40]. This supports the use of simple densitometry as an early quality check for heat-based thermal ablation.

Radiomics builds on this concept by quantifying texture heterogeneity that cannot be captured by margins or HU changes alone. In a retrospective study of 50 lung tumors treated with MWA, immediate post-procedure non-contrast CT texture features predicted 1-year LTP with greater sensitivity and specificity than contrast-enhanced CT at 4–6 week follow-up [40]. This approach could fundamentally shift the assessment of treatment adequacy from delayed follow-up imaging to intraprocedural assessment, enabling same-session re-ablation of undertreated lesions rather than surveillance for recurrence.

These metrics remain investigational, and densitometry and radiomic thresholds have not been standardized. Current studies are retrospective with small sample sizes; prospective studies that incorporate these immediate post-ablation imaging metrics into treatment algorithms are needed to determine if they reliably identify lesions that require additional same-session ablation, earlier surveillance, or an alternative treatment [40].

## 5. Expanding Applications

### 5.1. Larger Tumors and Central Lesions

Although current guidelines recommend percutaneous IGTA primarily for peripherally located tumors ≤ 3 cm, a subset of patients with larger or more central tumors are medically inoperable, have limited pulmonary reserve, or have exhausted radiation options; for these individuals, thermal ablation may represent the only remaining local therapy [3,5,35,41]. Clinical indications and patient selection for minimally invasive locoregional treatment are nuanced and depend on comorbidities and lesion characteristics, as summarized in Table 2.

Recent advances discussed in this review, including optimized energy delivery protocols, computational ablation zone modeling, intraprocedural navigation, and intraprocedural margin assessment, have improved margin confidence and technical precision. These developments are enabling treatment of tumors that approach or exceed conventional size thresholds. Nonetheless, tumor size remains a dominant predictor of local control across modalities; in the previously discussed cryoablation meta-analysis, tumor size was the only variable independently associated with 1-year LTC [16]. Power modulation in MWA may partially mitigate size-related limitations, as seen in the MALT trial, where size- and histology-based power protocols did not show increased LTP in larger tumors up to 4 cm [14]. Combination approaches with SBRT have also been explored. In a retrospective cohort of medically inoperable stage I NSCLC patients with 2–4 cm tumors, combined SBRT and cryoablation demonstrated improved long-term survival relative to SBRT-only cohorts, although direct comparison is limited by single-arm designs [23].

Central lung tumors present a different challenge, as proximity to the bronchovascular bundle is independently associated with increased local progression following MWA and higher procedural risk [45]. Adjunctive techniques such as artificial pneumothorax or hydrothorax can displace critical mediastinal structures to facilitate safe margins, though evidence is limited to a small cohort [46]. Cryoablation offers a specific advantage in this setting because real-time ice ball visualization allows for continuous monitoring of ablation extent adjacent to critical structures [4,16]. When combined with previously discussed navigation technologies, these approaches may permit treatment of previously inaccessible lesions.

Despite these advances, the evidence supporting IGTA use beyond current size and location constraints is limited, and until robust long-term outcome data are available, such applications should be reserved for carefully selected patients at experienced centers.

### 5.2. Repeat Ablation for Oligoprogression

A defining clinical advantage of percutaneous IGTA over surgery and SBRT is repeatability. Repeat surgical resection is technically challenging due to adhesions and results in permanent lung parenchyma loss, while retreatment with SBRT is constrained by cumulative dose, overlapping radiation fields, and progressive decline in lung function [35]. In contrast, percutaneous IGTA can safely be repeated with minimal impact on pulmonary reserve and is therefore particularly well suited for oligoprogression, defined as progression at a limited number (often ≤3) of sites while other disease sites remain controlled on systemic therapy [3,35].

The clinical benefit of this repeatability is illustrated in a multicenter retrospective study of 135 pulmonary oligorecurrences following prior radical surgical resection for NSCLC [18]. Over a median follow-up period of 31.8 months, 14.6% of patients developed local recurrence at the initial ablation site, and 43.7% showed new intrathoracic oligorecurrences. All local recurrences and 93% of the new intrathoracic lesions were successfully managed with repeated ablation. Importantly, local recurrence and intrathoracic oligorecurrences were not predictors of overall survival (OS) on multivariable analysis; only development of distant metastasis adversely affected survival [18]. These findings reframe the clinical role of ablation in oligoprogression as an ongoing local control strategy rather than a definitive curative treatment. As systemic therapies continue to extend survival in advanced NSCLC, the need for repeatable, lung-sparing local treatments of oligoprogression will grow.

### 5.3. Salvage After Prior Local Therapy

**Figure 2:**
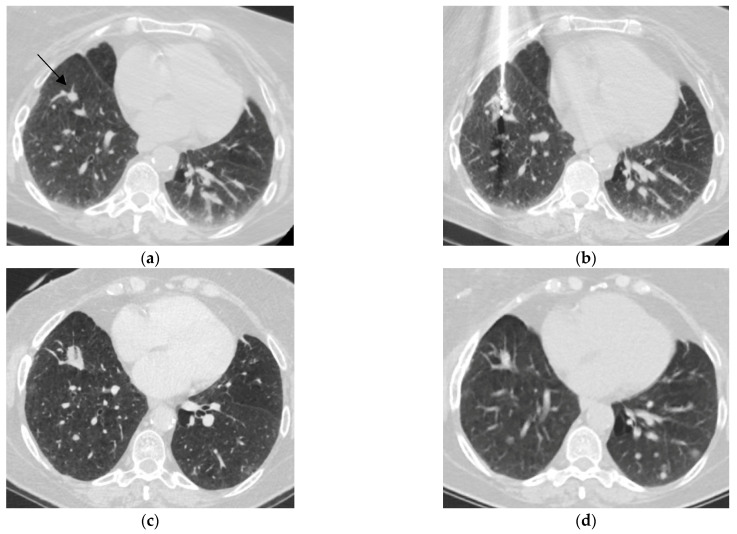
CT-guided percutaneous microwave ablation (MWA) of right lower lobe adenocarcinoma in a previously resected and irradiated patient. A 77-year-old female with stage IB NSCLC status post-two robotic right lower lobe (RLL) wedge resections and mediastinal lymph node dissection presented 20 months later with new pulmonary nodules, confirmed as biopsy-proven invasive adenocarcinoma with endobronchial ultrasound. Given prior thoracic radiotherapy to the right breast and regional lymph nodes two decades ago, stereotactic body radiotherapy (SBRT) was avoided to limit lung function loss, and percutaneous thermal ablation was pursued for treatment. MWA was chosen due to small lesion size and its proximity to vascular structures. (**a**) Non-contrast CT shows 1 cm RLL lesion (arrow). (**b**) A 15 G MWA antennae was advanced into the target lesion. Ablation was performed at 65 W for 3 min. (**c**) A 1-month CT scan shows expected post-ablation changes at the treatment site. (**d**) A 4-month CT scan shows evolution of the ablation zone with decreased consolidation and no evidence of local recurrence.

Thermal ablation has an established and expanding role as salvage therapy for local recurrence after SBRT. Approximately 10% of patients treated with curative-intent SBRT for medically inoperable early-stage NSCLC develop local recurrence [20,42]. Repeat radiation is often constrained by risk of radiation-induced lung injury [20,42]. The 2025 NCCN guidelines recognize percutaneous IGTA as a salvage option in this setting and emphasize multidisciplinary evaluation, including interventional radiology, when radiation fails [3]. A representative case of CT-guided percutaneous MWA as a salvage local therapy for recurrent right lower lobe adenocarcinoma following two prior wedge resections is shown in Figure 2.

Cryoablation is particularly well suited for salvage because it preserves surrounding lung architecture and can safely treat previously irradiated or fibrotic parenchyma. In a retrospective series of 29 patients with NSCLC recurrence after SBRT, repeat cryoablation achieved 100% technical success and a two-year OS of 62.3%, with minimal impact on pulmonary function despite 92.9% of the cohort having underlying chronic obstructive pulmonary disease (COPD) or emphysema [19]. Comparable results have been reported for MWA. A large study of 40 patients with stage I-IV NSCLC post-SBRT recurrence (average tumor size 2.4 ± 1.9 cm) showed 100% technical success and median OS of 51.0 months [20]. The consistency of these results across ablation modality and tumor characteristics in patients with significant baseline lung disease supports percutaneous IGTA as a parenchymal-sparing salvage option when repeat radiation is not feasible [19].

## 6. Emerging Clinical Data and Outcomes

Recent systematic reviews and meta-analyses provide a comprehensive assessment of percutaneous IGTA efficacy in early-stage NSCLC and pulmonary metastases. For stage I NSCLC, pooled 1-, 2-, 3-, and 5-year OSs were 96%, 81%, 68%, and 42%, respectively, with corresponding cancer-specific survivals (CSSs) of 98%, 88%, 75%, and 58% [47]. An important consideration is that the gap between OS and CSS at five years (42% versus 58%) may reflect the comorbidity burden of the typical ablation cohorts, which includes older patients with more severe baseline pulmonary disease or treatment-related lung injury. In this population, non-cancer mortality substantially influences OS. For example, patients with severe COPD and no lung cancer have an estimated 5-year survival of 40% [47]. CSS may therefore be a more accurate reflection of ablation efficacy in certain cohorts and should be considered alongside OS when evaluating outcomes.

Cryoablation shows comparable local control, with a pooled 1-year LTC of 90.5% (95% CI, 85.1–94.1%) in a meta-analysis of primary and metastatic tumors [16]. A recent retrospective analysis of 176 stage IA NSCLC patients treated with a triple-freeze–thaw protocol reported OS of 100% at one year and 94.7% at three years, underscoring the potential of durable disease control, especially with protocol optimization [17]. For pulmonary metastases, where prognosis is largely determined by systemic disease burden, a met-analysis reported pooled OSs of 92%, 68%, and 51% at 1, 3, and 5 years, with a 1-year LTC of 91% [48].

Given that ablation is uniquely repeatable with little effect on lung function compared with SBRT and surgery, secondary ablation outcomes after recurrence are an important consideration when evaluating the role of IGTA in lung cancer treatment. After recurrence following MWA for stage I NSCLC, secondary ablation achieved 1-, 2-, 3-, and 5-year OSs of 96.4%, 69.5%, 60.6%, and 26.1% [47]. For pulmonary metastases, secondary local effectiveness after repeat ablation was found to be 91.9–100%, exceeding primary LTC rates in the same cohort [48].

These outcomes position percutaneous IGTA as a durable local therapy with comparable long-term survival in early-stage NSCLC and pulmonary metastases, even in patients with substantial comorbidities. However, data are mostly derived from retrospective cohorts, and direct comparison with SBRT outcomes is limited by patient selection in ablation and radiation study groups.

## 7. Advances in Transarterial Chemoembolization

While percutaneous IGTA is used for early-stage, local recurrence, and oligoprogressive lung cancer, BACE fills a distinct clinical role for patients with stage IIIB or greater disease who have exhausted or cannot tolerate standard options. In this population, goals shift from local cure to symptom-directed therapy where controlling tumor burden may improve quality of life (QOL) [21,43].

Intravascular locoregional chemotherapy has been explored via both pulmonary and bronchial arterial system approaches for advanced or refractory lung cancer. In transpulmonary chemoembolization (TPCE), venous access is obtained to selectively catheterize tumor-supplying pulmonary artery branches. Chemotherapy mixed with lipiodol and, in some cases, additional embolic agents is then delivered to the tumor [49]. Initial series have demonstrated technical feasibility, local tumor control, and decreased systemic chemotherapy exposure [49].

**Figure 3:**
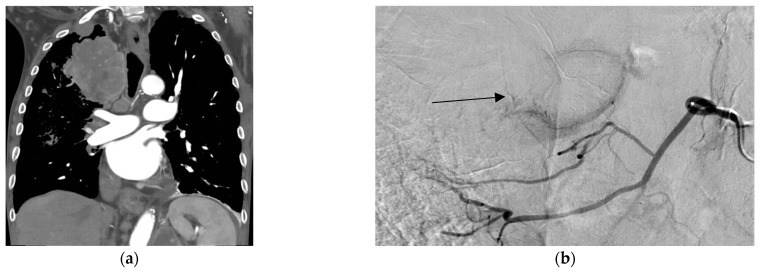
CT and selective angiography of right apical lung tumor in a patient with hemoptysis. A 63-year-old male presented with hemoptysis from a right upper lobe (RUL) mass. (**a**) Coronal CT of the chest shows a large necrotic right suprahilar mass. (**b**) Selective right bronchial arteriogram demonstrates tumor blush (arrow).

BACE builds on this concept by adapting the established bronchial artery embolization technique used for hemoptysis with intra-arterial chemotherapy infusion. When performed with drug-eluting beads (DEB-BACE), chemotherapy is delivered directly to lung tumors while simultaneously embolizing feeding vessels [43]. Figure 3 shows contrast-enhanced CT scans of a right apical lung tumor and selective bronchial angiogram with tumor blush, illustrating the targets typically treated with BACE.

Early BACE experience was limited by incomplete and non-targeted embolization causing complications, most notably spinal cord injury from embolization of radiculomedullary arteries [43]. Advances in angiographic mapping and superselective microcatheters have improved both safety and efficacy. Zhao et al. described advancing the microcatheter at least 2 cm distal to any potential spinal artery origin before embolization and reported no spinal cord injury or cerebral emboli in a multicenter prospective study of DEB-BACE for stage III-IV treatment refractory NSCLC [21]. The main AEs in the study were transient chest pain and low-grade fever [21].

In this same prospective series, median OS was 11.5 months, with 95% of patients achieving partial response or stable disease by modified response evaluation criteria in solid tumors (mRECIST) at two months. QOL also improved significantly across a range of symptoms including fatigue, dyspnea, insomnia, and physical functioning [21]. For patients who have no other therapy options, these functional improvements are clinically meaningful, and future BACE studies should incorporate QOL as a primary endpoint.

Current evidence for BACE is limited to small, single-center cohorts with varied treatment regimens and short follow-up. Large randomized or well-controlled comparative studies with consistent protocols are needed to establish patient selection for BACE and support its inclusion in lung cancer treatment guidelines.

## 8. Combined and Multimodality Strategies

As systemic therapies extend survival in NSCLC, a growing number of patients develop treatment resistance or cumulative toxicity that limits continued use. Others achieve partial response with oligoresidual disease. These clinical scenarios have created an interest in locoregional therapies as adjuncts to immunotherapy, radiation, and chemotherapy.

### 8.1. Ablation and Immunotherapy

Immune checkpoint inhibitors (ICIs) targeting programmed death-1 (PD-1) and programmed death ligand-1 (PD-L1) have become a central element of systemic therapy for advanced NSCLC, restoring anti-tumor cytotoxic T cell activity and reversing immune system suppression [22,44]. Thermal ablation induces cell death that releases tumor neoantigens and damage-associated molecular patterns. The biological rationale for an ablation–immunotherapy combination is that this process can amplify the systemic anti-tumor immune response when combined with ICI [22]. Two randomized trials provide early support for this approach.

In a single-center open-label randomized controlled trial of stage IIIB-IV NSCLC, cryoablation combined with PD-1 inhibitor camrelizumab was compared with camrelizumab plus platinum-based chemotherapy [22]. The cryoablation group received no chemotherapy yet achieved significantly longer median progression-free survival (PFS) (9.6 versus 8.3 months) and superior OS at one-year follow up, with a comparable safety profile [22]. There was evidence of potentiated immune system response in the study group with a statistically significant increase in CD4+/CD8+ ratio after four cycles.

The phase II BOOSTER trial provides the strongest evidence to date for ablation as local consolidative therapy to prolong the response of ICI in patients with oligoresidual disease. In this study, 62 patients with advanced NSCLC who developed oligoresidual disease after PD-1/L1 therapy were randomized to local consolidative ablation (heat-based and cryoablation) with continued immunotherapy versus immunotherapy maintenance alone [44]. Ablation combined with continued immunotherapy significantly prolonged PFS (median 26.7 vs. 11.7 months, *p* < 0.001), with a favorable OS trend (median survival not reached in the 17.8-month follow-up period). Ablation shifted treatment failure patterns towards progression at pre-existing lesions and reduced progression at new lesions when compared with maintenance immunotherapy alone, consistent with sustained systemic disease control [44]. An additional finding worth highlighting is that the subset of ablated tumors that had no viable disease on pre-ablation biopsy, representing complete response to prior immunotherapy, showed the highest PFS and OS. This suggests that thermal ablation is most effective as a consolidation in responsive oligoresidual disease rather than as salvage for treatment-resistant tumors [44].

These trials support two potential roles for ablation–immunotherapy combinations. In the first setting, cryoablation combined with PD-1 inhibitors showed superior outcomes compared with chemotherapy plus immunotherapy, raising the possibility of a chemotherapy-free approach to avoid myelosuppression while maintaining disease control [22]. In patients responding to immunotherapy, consolidative ablation of oligoresidual disease extended PFS, potentially eliminating residual viable tumor before it drives progression [44].

Both trials are limited by small sample sizes and short follow-up. Key questions remain, including optimal timing of ablation relative to immunotherapy, choice of ablation modality, and whether viable disease on pre-ablation biopsy should be a prerequisite for consolidative treatment. Larger trials are needed to define the role of IGTA combination approaches within immunotherapy treatment pathways.

### 8.2. Ablation and Radiation or Systemic Therapy

Combining IGTA with SBRT or chemotherapy theoretically leverages local tumor control provided by ablation to enhance the effectiveness of the primary systemic treatment while reducing its duration and cumulative toxicity. For inoperable stage I NSCLC, SBRT achieves > 90% local control, but OS is less favorable [23]. This may be because a radiologically occult viable tumor within a post-radiation scar may eventually lead to distant metastases. SBRT followed by percutaneous IGTA may represent a consolidative strategy to address this limitation. In a retrospective observational study of stage I NSCLC tumors ≥ 2 cm, SBRT followed by cryoablation within 3 weeks showed 3- and 5-year OSs of 87% and 74% [23]. Pulmonary function was preserved at 95% of pretreatment values, and there were no severe AEs. For comparison, SBRT alone for stage I tumors, including lesions < 2 cm, shows a 5-year OS rate of approximately 41–52% [23]. These results suggest that SBRT followed by IGTA may improve survival outcomes without a significant increase in pulmonary toxicity but must be interpreted cautiously given the single-arm design and comparison with previously published SBRT cohort data [23].

In advanced NSCLC, combining MWA with systemic chemotherapy may locally debulk the dominant lesions while systemic therapy controls metastatic disease at reduced cumulative dose and duration [24]. A 2025 meta-analysis found that MWA plus chemotherapy had improved PFS (pooled hazard ratio = 0.39, *p* < 0.001) and OS (pooled hazard ratio = 0.44, *p* < 0.001) with fewer chemotherapy-related AEs (pooled odds ratio = 0.62, *p* = 0.004) compared with chemotherapy alone [24]. The lower toxicity likely reflects a shorter treatment course with reduced cumulative chemotherapy dose in the combination group, facilitated by local tumor control with MWA. While these findings support combining MWA with chemotherapy to improve local control and survival outcomes with fewer side effects, the evidence base was limited to seven studies, of which only two were randomized controlled trials.

For all discussed IGTA combination strategies, a consistent finding is that adding ablation to systemic therapy or radiation appears to improve survival without increased AEs. However, the available evidence remains nascent and is derived from small, heterogeneous cohorts. Prospective trials designed to define optimal sequencing, patient selection, and long-term outcomes are essential before these strategies can be incorporated into multimodality treatment pathways.

### 8.3. Role of Transarterial Therapy in Multimodal Care

As with IGTA, early data suggest that BACE may enhance the effectiveness of systemic therapies while reducing toxicity in patients with advanced NSCLC refractory to or intolerance of standard treatments [25]. A single-center cohort comparing DEB-BACE/bronchial artery infusion chemotherapy (BAI) with and without PD-1 blockade found substantially longer median PFS (12.0 vs. 3.0 months, *p* < 0.001) and OS (27.0 vs. 8.0 months, *p* < 0.001) in the combination arm compared with BACE/BAI alone, with no increase in AEs [50]. In multivariable analysis, immunotherapy, tumor diameter < 6 cm, and at least two cycles of DEB-BACE/BAI were independently associated with longer PFS [50]. Notably, these favorable outcomes were observed in subjects 75 years and older with an ECOG of 3, suggesting that the combination was tolerable in patients traditionally considered poor candidates for additional therapy [50]. The non-randomized design, lack of immunotherapy control arm, and incomplete tumor PD-L1 data are important limitations, as imbalances in PD-L1 expression between groups may have contributed to the observed survival benefit. Similar findings were seen when DEB-BACE was combined with systemic chemotherapy for stage III-IV squamous cell carcinoma. The combination cohort achieved higher disease control (90.2% versus 61.2%, *p* = 0.003) and longer median OS (19 months versus 14 months, *p* = 0.015), with substantially lower rates of bone marrow suppression (7.3% versus 48.4%), despite identical systemic regimens [25].

These initial results from small non-randomized cohorts suggest that BACE as an adjunct to systemic therapies may extend disease control in specific patient populations. Larger prospective comparative trials with standardized BACE protocols and PD-L1 expression stratification are needed before this approach can be recommended in clinical practice.

## 9. Conclusions and Future Directions

This review evaluates recent advances in percutaneous IGTA and BACE for lung cancer, examining how technological developments can expand the range of treatable lesions and how emerging clinical data position these modalities as complements to surgery, SBRT, and systemic chemoimmunotherapy. The available evidence supports distinct clinical roles for each modality based on disease stage and patient characteristics.

For medically inoperable early-stage NSCLC when SBRT is not feasible, MWA and cryoablation are the primary locoregional options. The current data favor a default low-power approach in MWA with selective escalation and a triple freeze–thaw cryoablation protocol with active thaw [9,14,15,16,26]. Cryoablation is preferred near critical mediastinal structures, where real-time ice ball visualization permits continuous monitoring of ablation extent. It is also well suited for compromised lung parenchyma because it preserves tissue architecture, reducing post-procedure pulmonary function loss [4].

For oligoprogression in systemic therapy, IGTA is uniquely positioned as a repeatable lung-sparing modality. Retrospective data show that local and intrathoracic recurrences after initial ablation can be effectively re-ablated while maintaining favorable OS [18]. In the salvage setting after SBRT failure, both MWA and cryoablation demonstrated meaningful survival outcomes with minimal decrease in pulmonary function, even in patients with significant baseline lung disease [19,20,42]. For advanced treatment-refractory lung cancer, BACE has demonstrated promising short-term disease control and symptom relief [21].

The advances discussed in this review are expanding both the spectrum of tumors amenable to IGTA or BACE and the clinical context in which these modalities are used. Optimizing energy delivery protocols allows for the successful treatment of tumors that approach or exceed conventional size thresholds [14]. Improvements in intraprocedural navigation enable precise targeting of difficult-to-access lesions with fewer complications [14,31,33]. Computational modeling and intraprocedural imaging metrics may further improve local control through accurate margin prediction and real-time identification of undertreated lesions [30,40]. Early randomized data support combining IGTA or BACE with immunotherapy, chemotherapy, or radiation, with initial results demonstrating improved disease control without increased toxicity, support a possible role for this strategy in multimodal treatment pathways [22,23,24,44]. BACE is emerging as an option for disease control and symptom improvement in patients with advanced disease who have exhausted standard therapies [21,43].

There are gaps in the evidence and study limitations that have been acknowledged throughout this review. For IGTA, the relationship between ablation protocols, tumor-specific characteristics, and outcomes is unclear and requires further investigation through larger prospective randomized trials. Similarly, image-guided navigation technologies will need larger prospective and comparative studies demonstrating that improved lesion targeting translates to better long-term survival to allow for wider adoption. Computational ablation zone modeling and intraprocedural radiomic analysis require prospective validation. For BACE as a standalone therapy, establishing standardized treatment approaches and patient selection criteria through larger well-controlled studies is the next investigational step. Integrating IGTA/BACE into multimodality treatment pathways will require larger multicenter trials to establish the benefit seen in early randomized data and to define patient selection. With robust prospective multicenter and randomized studies, these percutaneous and transarterial modalities can transition from promising alternatives to evidence-based components in multimodal lung cancer treatment and, ultimately, have greater inclusion in guideline-based standards of practice.

## Figures and Tables

**Table 1:** Key recent studies in percutaneous image-guided thermal ablation (IGTA), bronchial artery chemoembolization (BACE), and combined treatment strategies for non-small cell lung cancer (NSCLC) and metastases. Outcome measures reflect terminology as reported in the original studies.

Study	Design	N (Patients)	Key Outcomes	Limitations	Conclusion
**Microwave Ablation (MWA)**					
Geevarghese et al. [7]	Retrospective single-center comparison of low (<60 W) versus standard (≥60 W) MWA in primary and metastatic lung tumors	145 (low power) 238 (standard power)	LCR 1-year 90.3% 2-year 84.7% Standard power associated with longer TTLR (sHR = 0.55 *p* = 0.003)	12-year enrollment with multiple operators, ablative margins not assessed, histology unconfirmed in some cases	Standard power had longer TTLR without more AEs when compared with low power
Liu et al. [9]	Retrospective multicenter propensity-weighted comparison of 40 W (default) versus 50 W (escalation) MWA in primary and metastatic lung tumors	409 (40 W default) 213 (50 W escalation)	Grade 3 AEs Default 5.1% Escalation 17.4% 1-year CAR Default 95.6% Escalation 90.6%	Confounding by indication (50 W reserved for incomplete margins or challenging cases), short follow-up	Default low power with selective escalation showed reduced AEs with modestly improved CAR
Iezzi et al. [14]	Prospective multicenter trial of standardized MWA power–time protocols based on tumor origin and size in primary and metastatic lung tumors	25 (primary) 29 (metastatic)	2-year LTP 28.6% (primary) 22% (metastatic)	Small sample size, short follow-up, no control group, heterogeneous tumor population	Pre-defined size-based power showed similar LTP across tumor sizes
Chan et al. [15]	Retrospective single-center evaluation of MWA safety, efficacy, and power–recurrence relationship in primary and metastatic lung tumors	93	Freedom from LR 1-year 87.6% 2-year 75.3% 3-year 69.2%	Small sample size, heterogeneous tumor population and treatment history with incomplete histologic confirmation	Power was not significantly associated with LR or complication rates
**Cryoablation**					
Sarshoghi et al. [16]	Systematic review and meta-analysis of 19 studies including primary and metastatic lung tumors	786	Pooled 1-year LTC 90.5% AE Incidence 4.9%	Observational studies only, short follow-up, heterogeneous protocols	Triple-freeze protocol associated with superior LTC and low AE rates on univariate analysis; small tumor size was the only predictor of LTC in multivariate analysis
Rehman et al. [17]	Retrospective single-center study of standardized triple freeze–thaw protocol for stage IA NSCLC	176	PFS 1-year 91.8% 3-year 89.4% OS 1-year 100.0% 3-year 94.7%	No control group, limited to stage IA tumors	Triple freeze–thaw cryoablation achieved excellent control and OS; local failures predominantly in tumors > 2 cm
**Oligoprogression and Salvage**					
Ni et al. [18]	Retrospective multicenter study of MWA for oligorecurrences after radical resection of NSCLC	103	LR 14.6% Median PFS 15.1 months Median OS 40.6 months	Heterogeneous adjuvant treatment, highly selected population, histology unconfirmed in some cases	MWA was effective and safe; prognosis driven by new intrathoracic/metastatic disease rather than LR
Fish et al. [19]	Retrospective single-center study of cryoablation of NSCLC recurrence after SBRT	29	PFS 1-year 70.8% 3-year 22.6% OS 1-year 92.9% 3-year 35.4%	Small sample size, heterogeneous use of systemic therapy, variation in cryoablation protocol	Salvage cryoablation for recurrence post-SBRT was feasible with preserved pulmonary function and acceptable PFS and OS
Mai et al. [20]	Retrospective single-center study of MWA and cryoablation of NSCLC recurrence after SBRT	40	Median PFS 15.7 months Median OS 51.0 months	Small sample size, heterogeneous use of systemic therapy post-ablation, mixed curative/palliative intent	Salvage IGTA for recurrence post-SBRT was safe and effective with grade 1 AEs
**Bronchial Artery Chemoembolization (BACE)**					
Zhao et al. [21]	Prospective multicenter study of DEB-BACE for refractory NSCLC	43	2-month DCR 95.35% Median OS 11.5 months	Small sample size, no control group, heterogeneous patient and tumor population	DEB-BACE showed high short-term control and symptom relief with grade 1–2 AEs
**Combined and Multimodal Strategies**					
Zheng et al. [22]	Randomized controlled trial, single-center comparing cryoablation + PD-1 inhibitor versus chemotherapy + PD-1 inhibitor in stage IIIB-IV NSCLC	60 (30 per arm)	Cryoablation + PD-1 inhibitor PFS 63.3% OS not reached Chemotherapy + PD-1 inhibitor PFS 43.3% OS 10.3 months	Small sample size, short follow-up, open-label design	Cryoablation + PD-1 inhibitors improved PFS, OS, and immune function compared with control group
Nomori et al. [23]	Retrospective multicenter study of SBRT followed by cryoablation in stage I NSCLC tumors ≥ 2 cm	64	5-year LCR 93% 5-year OS 74%	Small sample size, selection bias (56% chose treatment over surgery)	SBRT + cryoablation showed high 5-year LCR and OS
Lu et al. [24]	Systematic review and meta-analysis of MWA + chemotherapy versus chemotherapy alone	600	Combination group had better DCR (OR 2.48) and OS (HR = 0.44), with fewer AEs (OR 0.62)	7 studies with only 2 RCTs, chemotherapy regimen varied between studies	MWA + chemotherapy showed improved disease response, PFS, and OS without increased AEs
Lai et al. [25]	Retrospective single-center comparison of DEB-BACE + chemotherapy versus chemotherapy alone	41 (DEB-BACE + chemotherapy) 95 (chemotherapy only)	DEB-BACE + Chemotherapy DCR 90.2% Median OS 19 months Chemotherapy Only DCR 62.1% Median OS 14 months	Non-randomized, no immunotherapy limits generalizability	DEB-BACE + chemotherapy showed better DCR, PFS, and OS than chemotherapy alone

Abbreviations: AEs (adverse events); BACE (bronchial artery chemoembolization); CAR (complete ablation rate); DCR (disease control rate); DEB-BACE (drug-eluting bead bronchial artery chemoembolization); HR (hazard ratio); IGTA (image-guided thermal ablation); LCR (local control rate); LR (local recurrence); LTC (local tumor control); LTP (local tumor progression); MWA (microwave ablation); non-small cell lung cancer (NSCLC); OR (odds ratio); OS (overall survival); PFS (progression-free survival); RCT (randomized control trial); SBRT (stereotactic body radiotherapy); sHR (sub-hazard ratio); TTLR (time to local recurrence). Shaded rows indicate modality category headings.

**Table 2:** Clinical indications and patient selection for IGTA and BACE in lung cancer treatment.

Indication and Disease Stage	Key Considerations	Recommended Modality	References
Medically inoperable stage IA NSCLC (≤3 cm)	- Not a surgical candidate or refused surgery - Alternative local therapy to SBRT	IGTA	[3,5,34,35]
Medically inoperable stage IB-II NSCLC (>3 cm)	- Not a surgical candidate or refused surgery - Alternative to SBRT when contraindicated or refused - Multiple synchronous lesions requiring parenchymal preservation - Compromised pulmonary function	IGTA (cryoablation preferred for central lesions)	[3,4,14,16]
Oligoprogression in systemic therapy stage III-IV NSCLC	- ≤3 sites of progression while other disease remains controlled in systemic therapy - Cumulative radiation dose limits - Compromised pulmonary function	IGTA (repeatable with minimal impact on lung function)	[3,18,35]
Local recurrence after radiation or surgery, any disease stage	- Repeat surgery is not feasible - Cumulative radiation dose limitations or overlap with prior radiation fields - Compromised pulmonary function	IGTA (cryoablation for fibrotic lung parenchyma or central lesions)	[3,19,20,42]
Advanced treatment-refractory stage III-IV lung cancer	- Exhausted or intolerant of systemic therapy, SBRT, or additional surgery - Malignant hemoptysis or compressive symptoms	DEB-BACE (investigational)	[21,43]
Oligoresidual disease in immunotherapy stage III-IV NSCLC	- Residual viable disease at limited sites after initial response to immunotherapy	IGTA (investigational)	[22,44]

## Data Availability

No new data were created or analyzed in this study. Data sharing is not applicable to this article.

## Abbreviations

The following abbreviations are used in this manuscript:

AEs	Adverse Events
BACE	Bronchial Artery Chemoembolization
BAI	Bronchial Artery Infusion Chemotherapy
CAR	Complete Ablation Rate
CBCT	Cone Beam Computed Tomography
CI	Confidence Interval
COPD	Chronic Obstructive Pulmonary Disease
CSS	Cancer-Specific Survival
CT	Computed Tomography
DCR	Disease Control Rate
DEB-BACE	Drug-Eluting Bead Bronchial Artery Chemoembolization
DFS	Disease-Free Survival
ECOG	Eastern Cooperative Oncology Group
EMN	Electromagnetic Navigation
GGN	Ground-Glass Nodule
HR	Hazard Ratio
HUs	Hounsfield Units
IGTA	Image-Guided Thermal Ablation
ICIs	Immune Checkpoint Inhibitors
IPFA	Intraparenchymal Fine Needle Adjustment
LCR	Local Control Rate
LR	Local Recurrence
LTC	Local Tumor Control
LTP	Local Tumor Progression
MALT	Microwave Ablation Treatment of Lung Tumors
mRECIST	Modified Response Evaluation Criteria in Solid Tumors
MWA	Microwave Ablation
NCCN	National Comprehensive Cancer Network
NSCLC	Non-Small Cell Lung Cancer
OR	Odds Ratio
OS	Overall Survival
PD-1	Programmed Death-1
PD-L1	Programmed Death-Ligand 1
PFS	Progression-Free Survival
QOL	Quality of Life
RFA	Radiofrequency Ablation
SBRT	Stereotactic Body Radiotherapy
SCLC	Small Cell Lung Cancer
sHR	Sub-Hazard Ratio
TPCE	Transpulmonary Chemoembolization
TTLR	Time to Local Recurrence
